# The role of NADPH oxidase 1 in alcohol-induced oxidative stress injury of intestinal epithelial cells

**DOI:** 10.1007/s10565-022-09725-1

**Published:** 2022-05-31

**Authors:** Liuying Chen, Huikuan Chu, Lilin Hu, Zhonglin Li, Ling Yang, Xiaohua Hou

**Affiliations:** Division of Gastroenterology, Union Hospital, Tongji Medical College, Huazhong University of Science and Technology, 1277 Jiefang Avenue, Wuhan, 430022 China

**Keywords:** Alcoholic liver disease, Propionate, Apoptosis, Colonic epithelial cells, NADPH oxidase 1

## Abstract

Alcohol-mediated reactive oxygen species (ROS) play a vital role in intestinal barrier injury. However, the mechanism of ROS accumulation in enterocytes needs to be explored further. In our study, we found that chronic–binge ethanol-fed mice had increased levels of gut oxidative stress and high intestinal permeability. The transcription profiles of the colonic epithelial cells showed that the level of NADPH oxidase 1 (NOX1) was significantly elevated in alcohol-exposed mice compared with isocaloric-exposed mice. In vitro, NOX1 silencing alleviated ROS accumulation and the apoptosis of human colonic epithelial cells (NCM460), while NOX1 overexpression accelerated oxidative stress injury of NCM460 cells. Propionic acid was reduced in the gut of chronic–binge ethanol-fed mice, compared with isocaloric-fed mice, as observed through untargeted metabolomic analysis. Supplementation with propionate relieved ethanol-induced liver and intestinal barrier injuries and reduced the level of ROS accumulation and apoptosis of ethanol-induced colonic epithelial cells. Propionate alleviating NOX1 induced ROS injury of colonic epithelial cells, independent of G protein-coupled receptors. Propionate significantly inhibited histone deacetylase 2 (HDAC2) expressions both in ethanol-exposed colonic epithelial cells and TNF-α-treated NCM460. Chromatin immunoprecipitation (ChIP) assays showed that propionate suppressed the NOX1 expression by regulating histone acetylation in the gene promoter region. In conclusion, NOX1 induces oxidative stress injury of colonic epithelial cells in alcohol-related liver disease. Propionate, which can act as an endogenous HDAC2 inhibitor, can decrease levels of apoptosis of intestinal epithelial cells caused by oxidative stress.

**Graphical Abstract:**

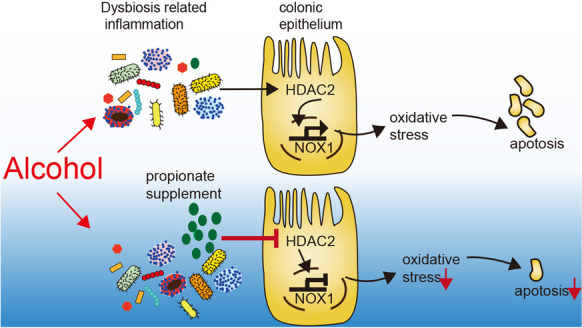

**Supplementary Information:**

The online version contains supplementary material available at 10.1007/s10565-022-09725-1.

## Introduction

The burden of alcohol liver disease (ALD) is globally growing, and effective treatment options are urgently required for patients with ALD (Louisa Degenhardt et al. 2018). Microbiota dysbiosis and injury of the intestinal barrier participate in the pathogenesis of ALD (Albillos et al. 2020). Studies have indicated that ethanol disrupts the tight junction of intestinal epithelial cells and causes the high permeability of the intestine (Bajaj 2019). Gut leakiness has been demonstrated in humans and mice with alcoholic liver disease (Hartmann et al. 2015). Restoration of the epithelial barrier function is a promising method of treatment for ALD since gut-derived microbial products and proinflammatory cytokines play a vital role in the progression of alcoholic steatohepatitis (Hartmann et al. 2015; Odenwald and Turner 2017).

Alcohol-mediated reactive oxygen species (ROS) cause the apoptosis of enterocytes and ubiquitin-dependent proteolytic degradation of junctional complex proteins (Cho et al. 2018). Intestinal cytochrome P450 2E1 (CYP2E1) is involved in alcohol-related intestinal oxidative stress injury and intestinal high permeability. Studies have shown that *Cyp2e1*-null mice were resistant to alcohol-induced gut leakiness (Abdelmegeed et al. 2013), while *Cyp2e1* inhibition by siRNA dramatically decreases levels of ROS in alcohol-exposed Caco-2 cells (Forsyth et al. 2013). The nicotinamide adenine dinucleotide phosphate (NADPH) oxidase (NOX) systems are the main enzymatic reactions that generate ROS in the gastrointestinal tract and are associated with inflammatory and oxidative stress responses (Aviello and Knaus 2018; Fink 2003). Compared with wild-type mice, NADPH oxidase–deficient (p47^phox–/–^) mice fed with a chronic ethanol diet had decreased levels of liver steatosis, inflammation, and necrosis; meanwhile, the production of free radical adducts in the liver was blocked (Kono et al. 2000). Pharmacological inhibition of NOX4 ameliorated alcohol-induced liver injury and reversed hepatic ROS accumulation (Sun et al. 2017). However, the role of NOX in alcohol-related gut ROS injury has not been fully elucidated.

Among the NOXs, NOX1 is particularly highly expressed in colonic epithelial cells. We found that NOX1 expression was obviously upregulated in the colonic epithelial cells of Lieber-DeCarli ethanol diet-fed mice, as shown through transcriptome sequencing. NOX1 has been found to participate in high-fat, diet-induced protein oxidation and the activation of redox-sensitive NF-κB and ERK1/2 pathways in the ileum of the mice (Cremonini et al. 2018). NOX1 mediated ROS injury of colonic epithelial cells in chronic DSS colitis mice, while NADPH oxidase inhibitors exerted a protective effect against the proinflammatory response of LPS in mouse colon epithelial cells during chronic DSS colitis (Ramonaite et al. 2014). In the current research, we investigated the mechanism of NOX1 affecting alcohol-associated intestinal barrier dysfunction.

NOXs expression is regulated by histone acetylation modification (Chen et al. 2016; Manea et al. 2018; Pietruczuk et al. 2019; Zelko and Folz 2015). Short-chain fatty acids (SCFAs) (including acetate, propionate, and butyrate) are key bacterial metabolites that provide nutrition to epithelial cells and regulate gut immune homeostasis (Chun et al. 2019; Koh et al. 2016). They are endogenous histone deacetylase (HDACs) inhibitors that are involved in the epigenetic regulation of targeted gene expression (Kim et al. 2016). Butyrate has been found to promote the transcription of tight junction proteins claudin-1, 3, and 4 (Wang et al. 2012; Yan and Ajuwon 2017), or by facilitating tight junction assembly (Peng et al. 2009), and then enhances intestinal epithelial barrier function. We aimed to determine whether SCFAs could reduce oxidative stress damage to the colon by regulating NOX1 expression.

Overall, our study aimed (1) to explore the role of NOX1 in alcohol-related ROS injury of enterocytes and (2) to investigate the protective effect exerted and the mechanism by which SCFAs alleviate oxidative stress injury in the colon.

## Materials and methods

### Animal treatment

Male C57BL/6J mice (8 weeks) were purchased from Vital River Labs (Beijing, China). The alcoholic liver injury model was constructed based on previous studies (Bertola et al. 2013). After 1 week of acclimatization, the mice adapted to a liquid diet for 5 days and were then subjected to a Lieber-DeCarli ethanol liquid diet for another 10 days. On the 16th day, mice were intragastric with a large dose of ethanol (5 g/kg body weight). For propionate treatment, mice were orally administered sodium propionate (Pro) (Sigma, St Louis, USA) dissolved in distilled water at a dose of 250 mg/kg body weight once daily during the ethanol liquid diet period. The control group was administered an equal volume of distilled water (ddH_2_O). The experiments were approved by the Institutional Research Ethics Committee of Union Hospital, Tongji Medical College, Huazhong University of Science and Technology.

### TUNEL staining

Paraffin-embedded colon tissues collected from control and treated mice were sliced into 4-μm slices. NCM460 cells were seeded onto glass slides and treated using TNF-α and sodium propionate, based on the work scheme. In situ apoptosis detection was performed using a TUNEL staining kit by following the manufacturer’s instructions (G1501, Servicebio, Wuhan, China). The slices were deparaffinized and washed using PBS. Then cells were fixed using 4% paraformaldehyde for 10 min. After being incubated with a proteinase K working solution and Triton X-100 (0.1%) for 10 min, TUNEL staining buffers were added and incubated for 1 h at 37 ℃. Finally, DAPI was used to visualize the nuclei.

### DCFH-DA and DHE staining

Colon tissues were embedded by OCT and sliced at 10 μm. Frozen colon tissues and NCM460 cells were stained using DCFH-DA (10 μM) and DHE (10 μM) according to the previous method for 30 min (Boonyong et al. 2020).

### Detection of oxidized proteins

Colonic epithelial cells were isolated from different groups of mice. The oxidized proteins were measured following the manufacturer’s instructions by a protein carbonyl assay kit (ab178020, Abcam, Cambridge, UK).

### Isolation of mice colonic epithelial cells

Colonic epithelial cells obtained from the control mice, ethanol-fed mice, and ethanol-fed sodium propionate-supplemented mice were isolated according to previously described protocols (Nik and Carlsson 2013). The entire colon was cut into 5-cm long sections, inverted, and washed using D-PBS to completely remove mucus and gut contents. One end of the inverted colon was ligated, and the air was injected from the other end. Then, the colon was submerged in 8 mmol/L EDTA for 5 min at 4 ℃. The epithelium layers were scraped and collected (500 g for 5 min). Total RNA and protein were extracted for subsequent use.

### mRNA sequencing

RNA sequencing of isolated colonic epithelial cell libraries was performed by Personal Bio Biotechnology (Shanghai, China) using an Illumina platform. Analysis of differentially expressed genes (DEGs) was done by DESeq2. Genes with a *P* value of < 0.05 and |log_2_(fold change)|> 1.5 were identified as DEGs. Gene ontology (GO) analysis and Kyoto Encyclopedia of Genes and Genomes (KEGG) pathway analysis were conducted to analyze the biological functions of DEGs using the bioinformatics tool, NetworkAnalyst.

### Liver injury and endotoxin measurement

Paraffin-embedded liver tissues were sliced into 4-μm sections, then hematoxylin and eosin (H&E) staining was performed. OCT-embedded liver tissues were sliced (10 μm) and stained using oil red. Levels of serum alanine aminotransferase (ALT) and alanine aminotransferase (ALT) were measured by commercial kits (Nanjing Jiancheng Bioengineering Institute, Nanjing, China). Endotoxin levels were examined using an LPS ELISA kit (Solarbio, Beijing, China).

### Immunofluorescence and immunohistochemical staining

Paraffin-embedded colon tissues were sliced into 4-μm sections. After blocking using 5% bovine serum albumin for 1 h, the deparaffinized slices were incubated with the primary antibodies occludin (product #40–4700, 1:50; Invitrogen, California, USA), ZO-1 (product #61–7300, 1:25; Invitrogen, California, USA), claudin-1 (product #37–4900, 1:50; Invitrogen), and 4 hydroxynonenal (4-HNE) (product #GTX01087, 1:200; GeneTex, California, USA) overnight at 4 ℃. After washing, the Alexa Fluor 594 conjugated goat anti-Rabbit IgG (H + L) secondary antibody (product #8889, 1:500; CST, Massachusetts, USA) and Alexa Fluor 594 conjugated goat anti-Mouse IgG (H + L) secondary antibody (product #8890, 1:500, CST) were added for 1 h at room temperature. Then, DAPI was used to visualize the nuclei. For 4-HNE detection, the slices were incubated with HRP conjugated goat anti-Rabbit IgG (H + L) secondary antibody (1:200, product #G1215; Servicebio, Wuhan, China) for 1 h, then stained with DAB working solution. Nucleoli were stained with hematoxylin. Finally, the slides were observed using an Olympus microscope and a NIKO laser scanning confocal microscope.

### RT-PCR

TRIzol reagent (Invitrogen, California, USA) was used to extract total RNA from isolated colonic epithelial cells and NCM460 cells. Then, cDNA was synthesized with PrimeScript™ RT Master Mix (RR036A, TaKaRa) from 1 ug RNA. Thereafter, qPCR reactions were performed on the LightCycler 96 Amplifier (Roche Diagnostics, Mannheim, Germany) using PowerUp™ SYBR™ Green Master Mix (Invitrogen, Carlsbad, CA). The detection of liver translocation bacteria was based on previous methods (Hendrikx et al. 2019; Maeda et al. 2003). Primer sequences are shown in Supplementary Table 1.

### Immunoblotting

Primary colonic epithelial cell and NCM460 cell homogenates were created using RIPA buffer. Protein concentration was quantified by the BCA kit (P0010, Beyotime Biotechnology, Shanghai, China). An equal amount of protein (40 mg) was separated by 10% polyacrylamide gels and transferred to polyvinylidene fluoride membranes. Then the proteins were probed using the primary antibodies, Bax (1:1000, 50,599–2-Ig; Proteintech, Rosemont, USA), active and pro Caspase-3 (1:1000, A19654; ABclone, Wuhan, China), Caspase 8 (1:1000, 13,423–1-AP; Proteintech), iNOS (1:500, ab178945; Abcam, Cambridge, UK), NOX1 (1:500, ab131088), and HDAC2 (1:1000, ab124974). After the corresponding secondary antibodies were incubated, the bands were detected with ultra-sensitive ECL reagents (P0018FS; Beyotime Biotechnology, Shanghai, China).

### Cell culture and treatment

Human colonic epithelial cells, NCM460, were cultured in DMEM medium supplemented with 10% fetal bovine serum and 100 U/mL penicillin/streptomycin at 37 ℃ and 5% CO_2_. The NCM460 cells were treated using TNF-α (PeproTech, Cranbury, USA) at doses of 5 ng/mL and 10 ng/mL for 24 h to induce oxidative stress injury. To measure the protective effect exerted by sodium propionate, the NCM460 cells were treated with sodium propionate (4 mM and 8 Mm) for 2 h before being incubated with TNF-α for another 24 h.

To knock down the expression of NOX1, free fatty acid receptor (FFAR) 2, and FFAR3 in NCM460 cells, siRNAs were purchased from Sangon (Shanghai, China) and used as instructed by the manufacturer.

To overexpress NOX1 in NCM460 cells, lentivirus was purchased from Genechem (Shanghai, China). NCM460 cells transfected with lentivirus were screened with puromycin.

### Untargeted metabolomic analyses of cecal contents

The metabolomics of the cecal contents obtained from control and ethanol-fed mice were analyzed using ultra-high-performance liquid chromatography equipped with quadrupole time-of-flight mass spectrometry (UHPLC-QTOF/MS). The original data were processed for peak alignment, retention time correction, and peak area extraction by XCMS software. Then, principal component analysis (PCA), partial least square discriminant analysis (PLS‐DA), and Student’s *t*‐test analysis were performed. Metabolites with variable importance in the projection (VIP) value of > 1 in the PLS‐DA analysis and a *p* value of < 0.05 in Student’s *t*‐test were defined as significantly different.

### Chromatin immunoprecipitation (ChIP)

ChIP experiments were performed by the SimpleChIP® Enzymatic Chromatin IP Kit (product #9002; CST, Massachusetts, USA) as previously described (Lu et al. 2013). Chromatin samples that cross-linked with proteins were put to react with anti-HADC2 (ab124974, Abcam), anti-RNA polymerase II (ab238146, Abcam), anti-histone H3 (acetyl K9) (ab32129, Abcam), and anti-histone H3 (acetyl K27) (ab177178, Abcam). The precipitated genomic DNA was amplified using the primers of the NOX1 promotor region listed in Supplementary Table 2.

### Statistical analysis

The data are presented as mean ± mean standard error (SEM). Student’s *t*-test and Mann–Whitney test were used to determine differences between groups. *P* value of less than 0.05 was considered to indicate significance.

## Results

### Chronic–binge ethanol feeding mice showed increased gut oxidative stress and high intestinal permeability

**Fig. 1:**
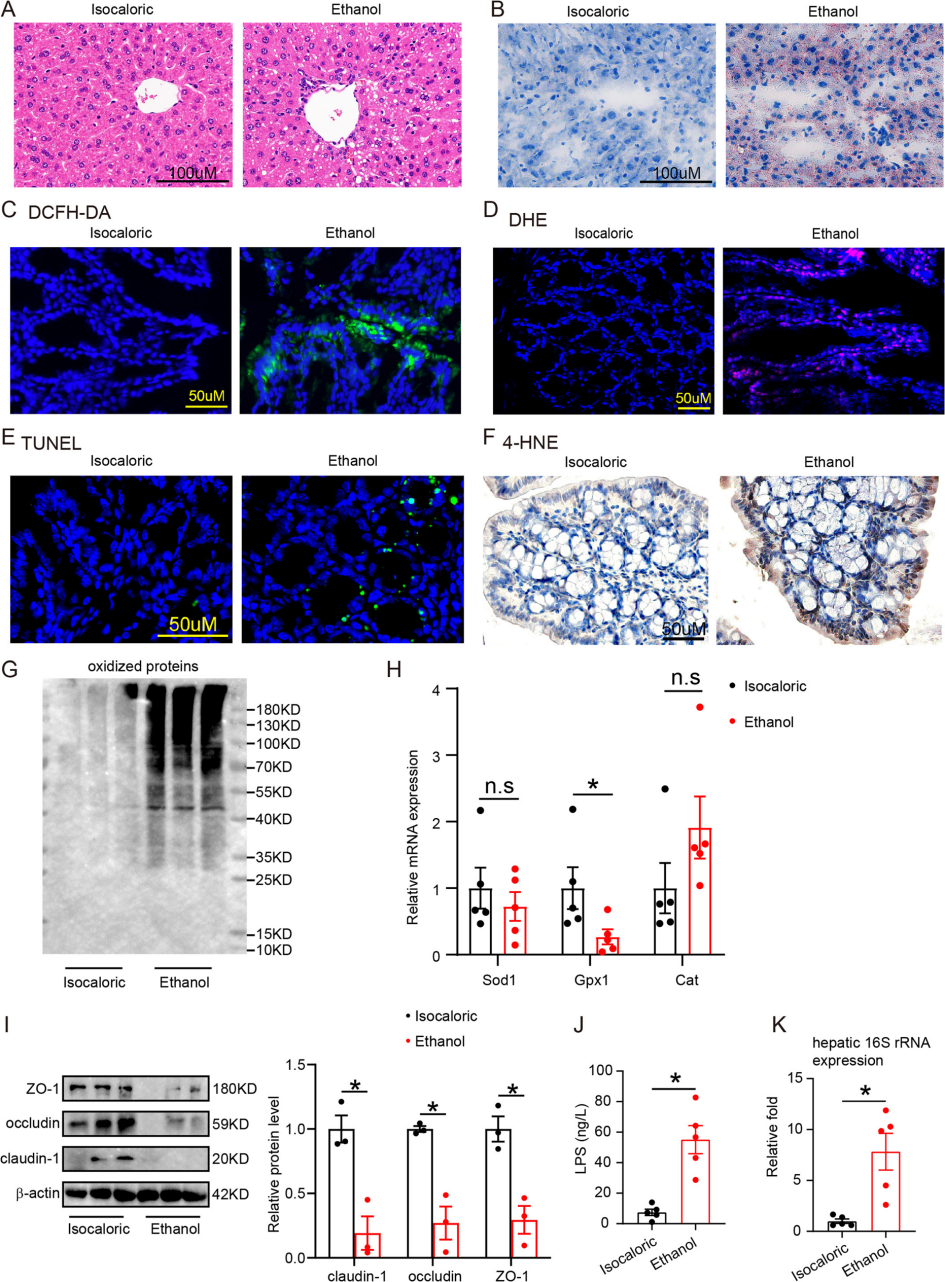
Chronic–binge ethanol feeding mice had increased oxidative stress and high intestinal permeability in the colon. **A**, Liver HE staining of chronic–binge ethanol feeding mice and isocaloric feeding mice. **B**, Liver oil red staining of chronic–binge ethanol feeding mice and isocaloric feeding mice. **C, D,** DCFH-DA fluorescent probe assay (**C**) and DHE staining (**D**) showed ROS formation in colonic epithelial cells of the above groups. **E**, TUNEL staining showed apoptotic cells in the colon of the above groups. **F**, 4-HEN staining detected lipid peroxidation in the colon of the above groups. **G**, Oxidized proteins in colonic epithelial cells of the above groups. **H**, Antioxidative factors Sod1, Gpx1, and Cat expression measured by RT-PCR. **I**, Immunoblotting tested tight junction proteins occludin, ZO-1, and claudin-1 expression in mice colonic epithelial cells of the above groups. **J**, Serum LPS levels of above groups. **K**, Hepatic 16S rRNA gene expression normalized to 18S rRNA of the above groups. n.s., no significant. *p value < 0.05

Chronic–binge ethanol-fed mice with liver lipid accumulation (Fig. 1A, B) had increased levels of reactive oxygen species accumulation in their colon epithelial cells, as measured using DCFH-DA (Fig. 1C), DHE (Fig. 1D), and 4-HNE (Fig. 1F) staining, compared with isocaloric-fed mice. The level of apoptosis of colon epithelial cells was higher in alcohol-exposed mice compared with control mice (Fig. 1E). Moreover, an increase in the level of oxidized proteins (Fig. 1G) and a decrease in the level of antioxidative factor Gpx1 expression (Fig. 1H) were found in chronic–binge alcohol-exposed mice compared with the controls. The levels of claudin-1, occludin, and ZO-1 (Fig. 1I) were decreased in chronic–binge alcohol-exposed mice in comparison with the controls. Serum LPS level (Fig. 1J) and hepatic bacterial translocation (Fig. 1K) increased significantly in chronic–binge alcohol-fed mice compared with the controls.

### mRNA sequencing was used to explore the mechanism of alcohol-related oxidative stress injury of the mice colon

**Fig. 2:**
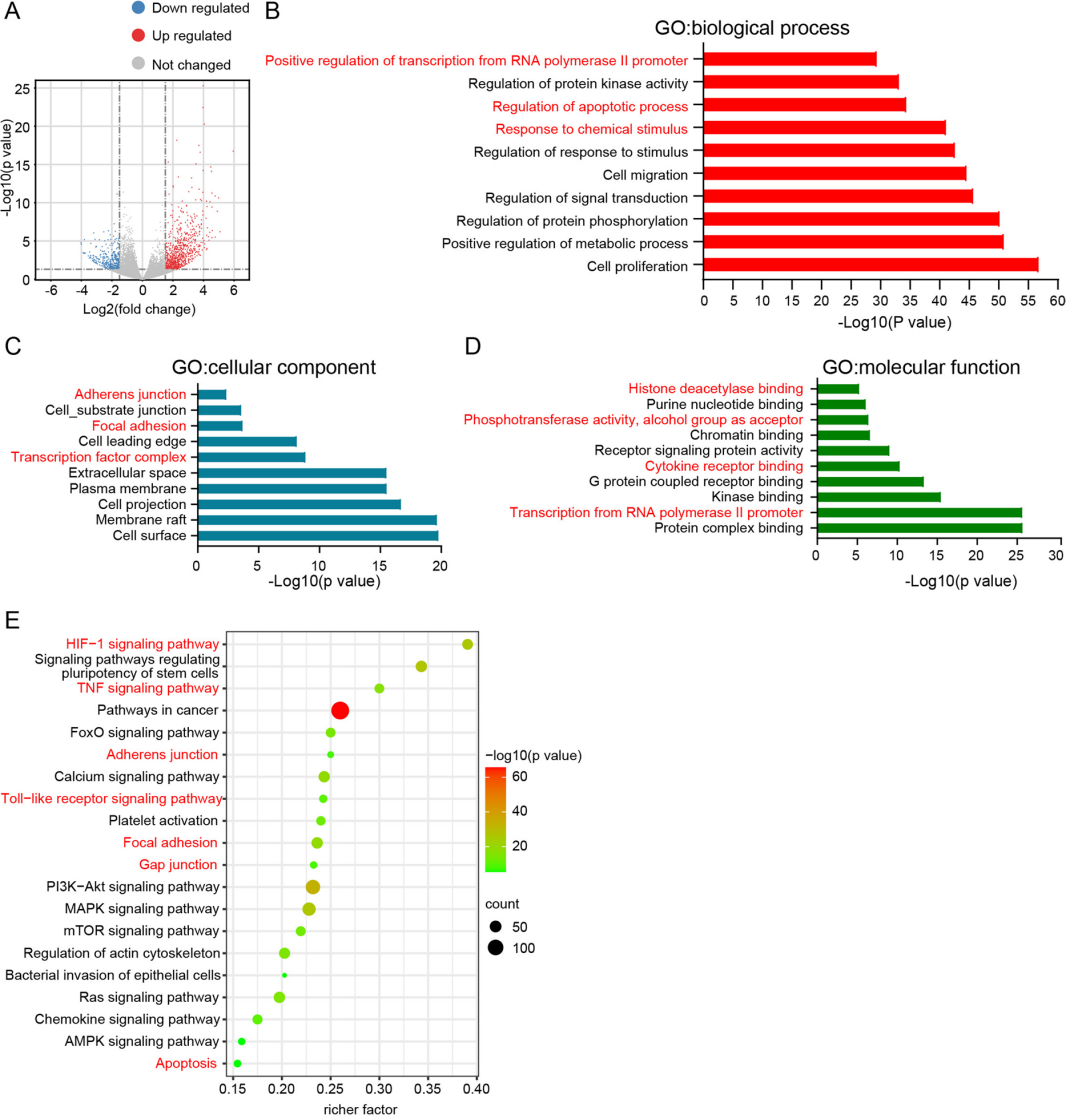
Transcription profiles analysis of colonic epithelial cells isolated from the control group and ethanol-fed group. **A**, Volcano figures illustrated differentially expressed genes (DEGs) between the control group and alcohol-fed group. |log_2_ (fold change)|> 1.5; *p* value < 0.05. **B**–**D**, GO functional enrichment analysis performed by DEGs. **E**, KEGG pathway analysis performed by DEGs. *N* = 3 for each group. *p value < 0.05

To determine the mechanism by which alcohol caused oxidative stress injury of the mice colon, we created the transcription profiles of colonic epithelial cells obtained from isocaloric-fed and alcohol-fed mice. As shown in Fig. 2A, 344 genes were downregulated and 759 genes were upregulated in the colonic epithelial cells of ethanol-fed mice compared with isocaloric-fed mice. GO functional enrichment and KEGG pathway analysis were conducted to determine the mechanism by which alcohol exerts its effects on colonic epithelial cells. As shown in Fig. 2B, under the biological process classification, the differential expression genes (DEGs) were enriched in positive regulation of transcription from RNA polymerase II promoter, regulation of the apoptotic process, and response to a chemical stimulus. Under the cellular component classification (Fig. 2C), the DEGs were enriched in adherens junction, focal adhesion, and transcription factor complex. Under the molecular function classification (Fig. 2D), the DEGs were enriched in histone deacetylase binding, phosphotransferase activity (alcohol group as acceptor), cytokine receptor binding, and transcription from RNA polymerase II promoter. The results of the KEGG pathway analysis were displayed using a scatterplot (Fig. 2E). The DEGs were clustered in the HIF − 1 signaling pathway, TNF signaling pathway, adherens junction, toll-like receptor signaling pathway, focal adhesion, gap junction, and apoptosis.

Similar to the results mentioned above, previous studies have indicated that dysbiosis-induced TNF-α signaling mediated the disruption of the intestinal barrier in alcohol-exposed mice (Chen et al. 2015). Therefore, we assumed that TNF-α signaling caused oxidative stress injury in colonic epithelial cells.

### NOX1 was responsible for ROS injury of colonic epithelial cells

**Fig. 3:**
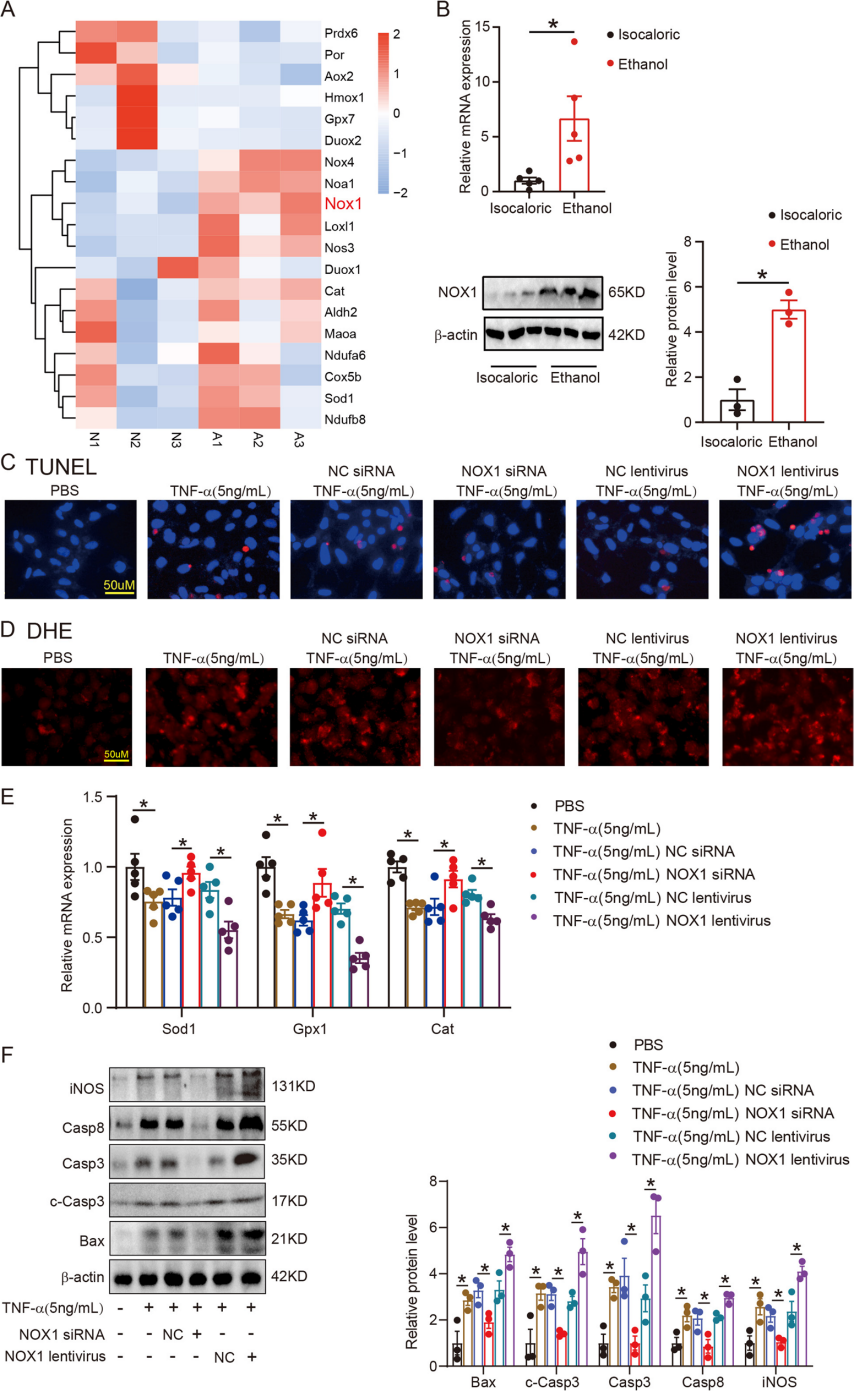
NOX1 was responsible for ROS injury of colonic epithelial cells. **A**, List of genes related to oxidative stress. **B**, NOX1 mRNA and protein levels of colonic epithelial cells isolated from isocaloric-fed and ethanol-fed mice were measured by RT-PCR and western blotting. **C**, TUNEL staining showed apoptosis of NOX1 silenced (by siRNA) and overexpressed (by lentivirus) NCM460 cells treated with TNF-α. **D**, DHE staining showed ROS formation in NOX1 silenced and overexpressed NCM460 cells treated with TNF-α. **E**, Antioxidative factors Sod1, Gpx1, and Cat expression measured by RT-PCR of above groups. **F**, Immunoblotting showed Bax, Casp3, c-Casp3, Casp8, and iNOS protein expressions of NCM460 cells in the above group. *N* = 3 for each group. *p value < 0.05

The analysis of genes associated with oxidative stress showed that NOX1 level was obviously elevated in alcohol-exposed mice compared with controls (Fig. 3A). The analysis of total RNA and protein extracts further verified the elevated expression of NOX1 in alcohol-exposed mice in comparison with controls (Fig. 3B). In vitro, both alcohol and TNF-α treatment induced high expression of NOX1 in normal human colonic epithelial (NCM460) cells (Supplementary Fig. 1). The RNA expression profile analysis showed that the TNF signaling pathway was implicated in alcohol-related intestinal barrier dysfunction (Fig. 2E). Gene expression of TNF-α was also significantly upregulated in colonic tissues of mice fed alcohol compared with control mice (Supplementary Fig. 2). Therefore, we only treated normal human colonic epithelial cells (NCM460) with TNF-α. TNF-α caused apoptosis and ROS accumulation in NCM460 cells, but this effect was abolished by the NOX1 silencing and enhanced by NOX1 overexpression (Fig. 3C, D and Supplementary Fig. 3). Expressions of antioxidative factors Sod1, Gpx1, and Cat were decreased in NCM460 cells treated with TNF-α, compared with PBC treatment, decreased after NOX1 silencing, and increased further after NOX1 overexpression (Fig. 3E). The results of the immunoblotting analysis were consistent with the immunofluorescence results. The protein level expression of Bax, Casp3, c-Casp3, Casp8, and iNOS increased in TNF-α-treated NCM460 cells, compared to the control, reduced following NOX1 silencing, and further elevated following NOX1 overexpression (Fig. 3F).

### Propionic acid was reduced in the gut of chronic–binge ethanol-fed mice

**Fig. 4:**
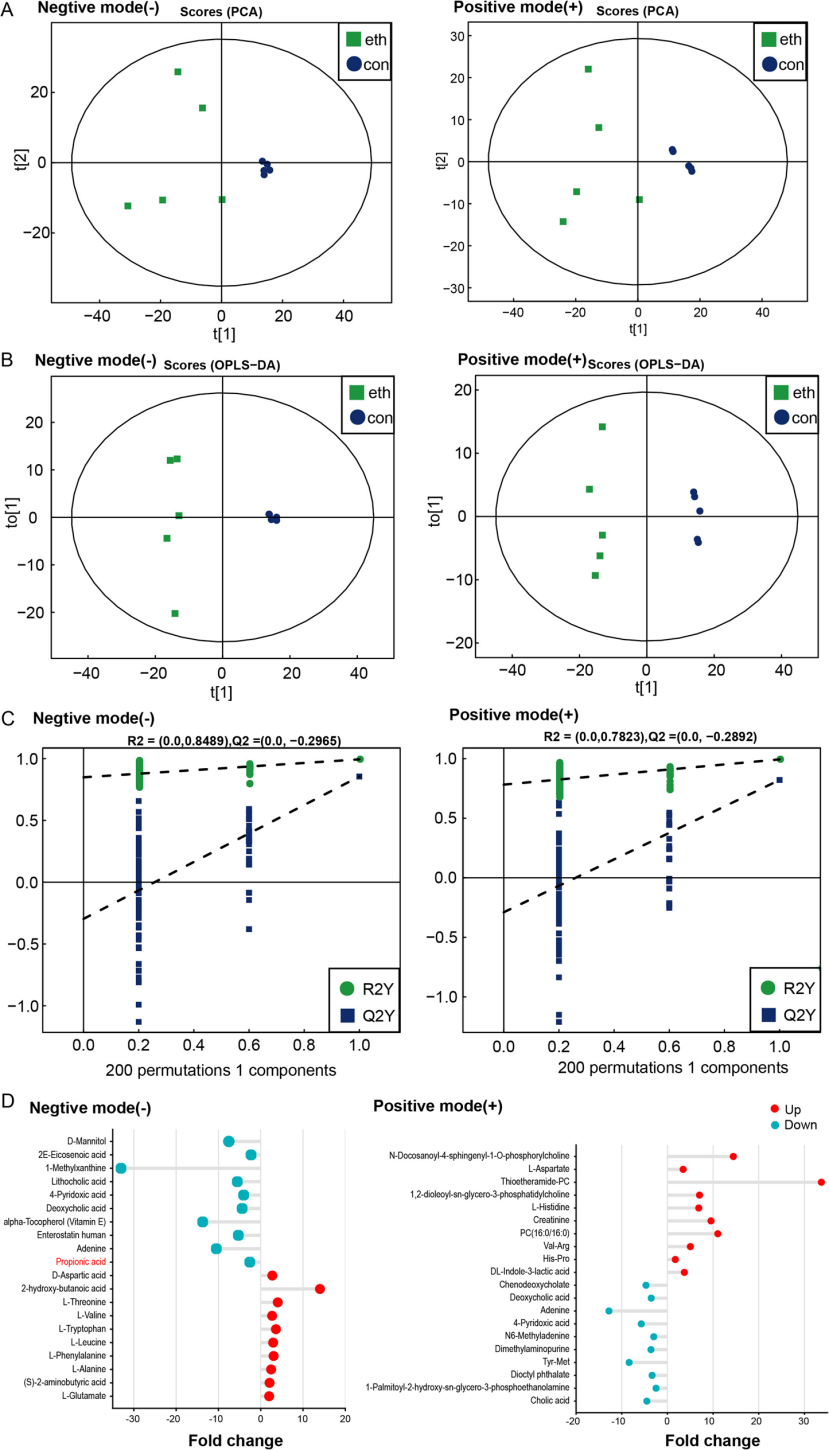
Metabolomic analysis of cecal contents from control and chronic–binge ethanol feeding mice. **A**, The principal component analysis (PCA) in negative ion mode and positive ion mode. **B**, The orthotopic partial least-squares discriminant analysis (OPLS-DA) score plots of positive ion mode and negative ion mode. **C**, Permutation tests for the OPLS-DA score plots. **D**, The top 10 changed metabolites of both ionization modes, compared with the control group and chronic–binge ethanol feeding group. eth: chronic–binge ethanol feeding group; con: control group. *N* = 5 for each group

Since modulation of the gut microbiota is a therapeutic target in alcohol-related liver disease (Sarin et al. 2019), we performed an untargeted metabolomic analysis of cecum contents obtained from isocaloric-fed and chronic–binge ethanol-fed mice. The PCA score plot shows a clear separation between isocaloric and ethanol samples under both ionization modes, indicating the presence of significant metabolic differences after chronic alcohol exposure (Fig. 4A). To further profile the differential metabolism, we performed an OPLS-DA analysis and permutation tests (Fig. 4B, C). The top 10 differential metabolites of both ionization modes are listed in Fig. 4D. Propionic acid levels decreased significantly in ethanol-fed mice compared with isocaloric-fed mice.

### Supplementation with propionate relieved ethanol-induced liver and intestinal barrier injury

**Fig. 5:**
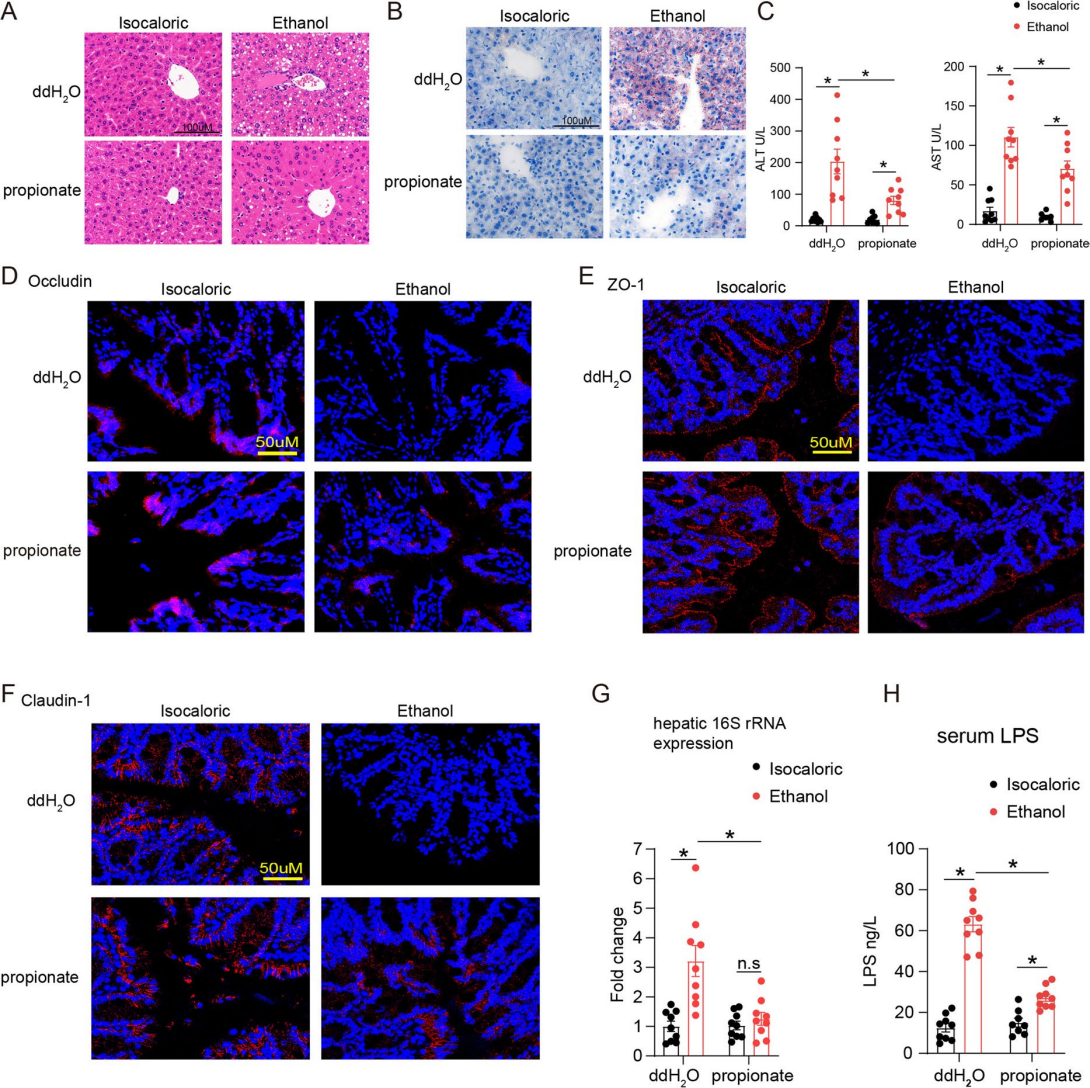
Propionate supplement protected ethanol-induced liver injury and intestinal high permeability. **A**, Representative images of liver HE staining of isocaloric feeding mice and chronic–binge ethanol feeding mice with and without propionate supplement. **B**, Representative images of liver oil red staining of above groups. **C**, Serum ALT and AST levels of the above groups. *N* = 9 for each group. **D–F**, Immunofluorescence tested tight junction proteins occludin (**D**), ZO-1 (**E**), and claudin-1 (**F**) expression in colonic tissues of the above mice groups. **G**, Hepatic 16S rRNA gene expression normalized to 18S rRNA. *N* = 9 for each group. **H**, Serum LPS levels by ELISA. *N* = 9 for each group. *p value < 0.05

Chronic–binge ethanol-fed mice showed obvious lipid deposits in the liver and a high serum level of ALT and AST (Fig. 5A–C). Propionate supplementation reduced the amount of lipid deposits and liver injury. Meanwhile, propionate administration predominantly relieved colon barrier impairment and endotoxemia. The expression of occludin, ZO-1, and claudin-1 was downregulated in the colon tissues of ethanol-fed mice compared with isocaloric-fed mice, while levels of these tight junction proteins increased after supplementation with propionate (Fig. 5D–F). Furthermore, an increased level of bacterial translocation in the liver and serum LPS was found in the ethanol-fed mice, compared with the isocaloric-fed mice, while levels decreased after supplementation with propionate (Fig. 5G, H).

### Propionate supplementation reduced ROS accumulation and apoptosis of ethanol-induced colonic epithelial cell

**Fig. 6:**
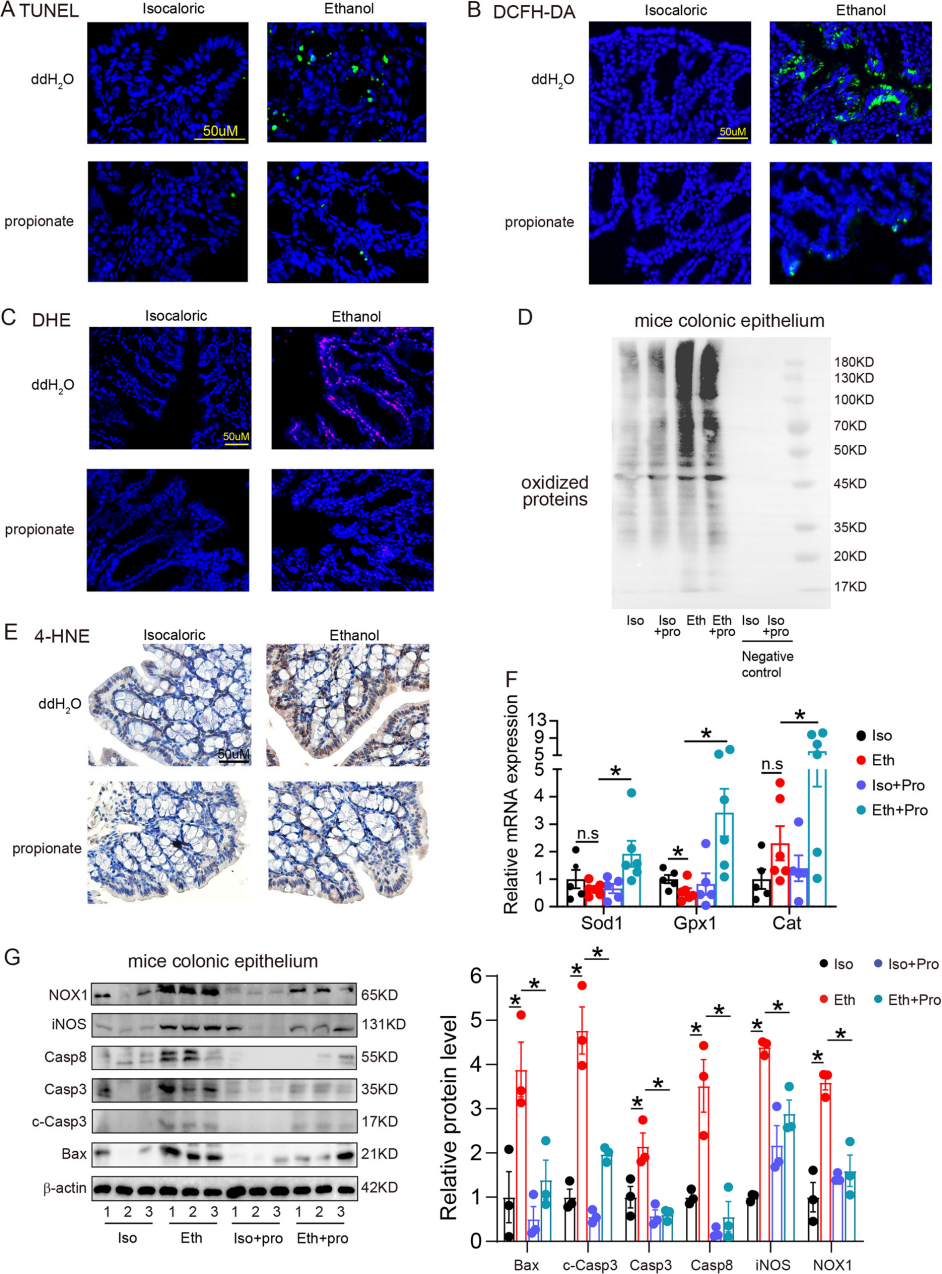
Propionate supplementation reduced ROS accumulation and apoptosis of ethanol-exposed colonic epithelial cells. **A**, TUNEL staining of colonic tissues from isocaloric-fed and ethanol-fed mice with and without propionate supplement. **B**, **C**, DCFH-DA fluorescent probe assay (**B**) and DHE staining (**C**) showed ROS formation in colonic tissues of the above groups. **D**, Oxidized proteins of colonic epithelial cells isolated from the above groups measured by immunoblotting. **E**, 4-HNE staining measured colonic lipid peroxidation of the above groups. **F**, RT-PCR evaluated the antioxidative factors Sod1, Gpx1, and Cat expressions of the above groups. **G**, Immunoblotting showed Bax, Casp3, c-Casp3, Casp8, iNOS, and NOX1 protein expressions of mice colonic epithelial cells isolated from the above groups. *N* = 3 for each group. n.s, no significant. *p value < 0.05. Iso: isocaloric feeding group; Eth: chronic–binge ethanol feeding group; pro: propionate

TUNEL positive colonic epithelial cells decreased significantly in ethanol-exposed mice treated with propionate compared with ethanol-exposed mice without propionate treatment (Fig. 6A). The DCFH-DA fluorescent probe assay and DHE staining showed intracellular ROS accumulation in the ethanol-exposed mice, which decreased after propionate supplementation (Fig. 6B, C). Additionally, the concentration of oxidized proteins and lipid peroxidation increased in the colonic epithelium of ethanol-exposed mice compared to the control mice (Fig. 6D, E). After propionate treatment was administered, the levels of the oxidized proteins and lipid peroxidation decreased compared with that of mice administered ddH_2_O. The enzymatic antioxidants Sod1, Gpx1, and Cat of colonic epithelial cells were significantly elevated in ethanol-exposed mice with propionate supplement compared with ddH_2_O supplement (Fig. 6F).

Immunoblotting analysis showed that the expression levels of Bax, Casp3, c-Casp3, Casp8, iNOS, and NOX1 were upregulated in the colonic epithelium of ethanol-exposed mice compared to control mice and were downregulated when supplemented with propionate (Fig. 6G). Meanwhile, immunofluorescence and immunoblot both stated that propionate prevented TNF-α caused ROS generation and the apoptosis of colonic epithelial cells in vitro (Supplementary Fig. 4). These results revealed that ROS-mediated apoptosis is critical for the propionate-mediated reduction of intestinal permeability.

### Propionate alleviates NOX1-induced ROS injury of colonic epithelial cells independent of G protein-coupled receptors

**Fig. 7:**
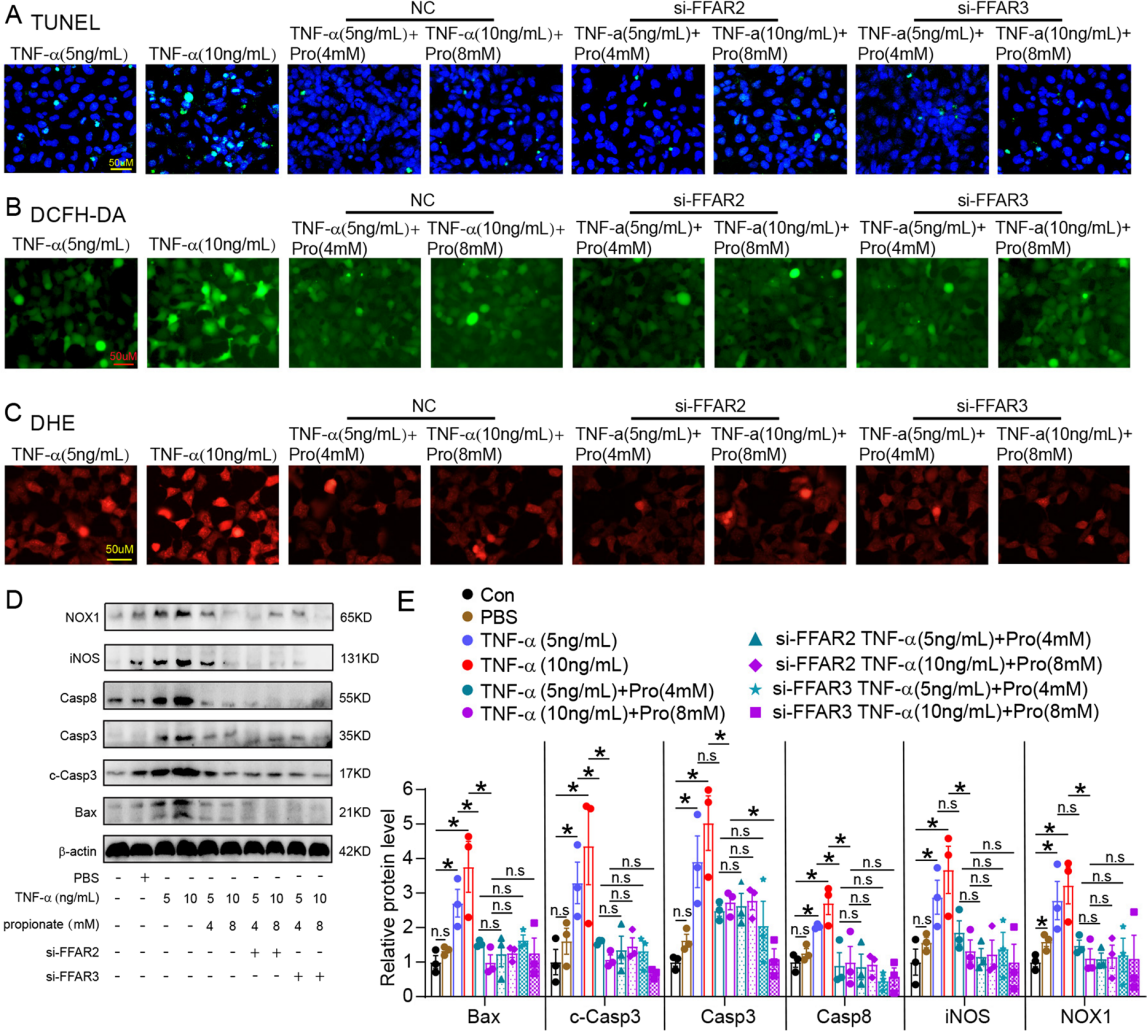
Propionate alleviating ROS injury of colonic epithelial cells was independent of G protein-coupled receptors. **A**, TUNEL-labeled apoptosis of WT and FFAR2 and FFAR3 knockdown NCM460 cells treated with TNF-α and propionate. **B, C**, DCFH-DA (**B**) and DHE staining (**C**) showed the ROS formation in NCM460 cells of the above-treated groups. **D, E**, Immunoblotting images (**D**) and quantification analysis (**E**) of Bax, Casp3, c-Casp3, Casp8, iNOS, and NOX1 protein expressions of the above-treated groups. n.s, no significant. *N* = 3 for each group. *p value < 0.05

We designed siRNA to intervene with propionate receptors FFAR2 and FFAR3 which have been found to be expressed in colonic epithelial cells and have a relatively high affinity to propionate (Koh et al. 2016). Knockdown of FFAR2 and FFAR3 expression was verified using RT-PCR and western blotting (Supplementary Fig. 5). As shown in Fig. 7A–C, propionate prevented TNF-α induced apoptosis and ROS generation in colonic epithelial cells, but no exacerbation of apoptosis and ROS damage was observed in NCM460 after FFAR2 and FFAR3 knockdown, as revealed by TUNEL, DCFH-DC, and DHE staining. The immunoblotting analysis showed that FFAR2 and FFAR3 knockdown were not induced to upregulate Bax, Casp3, c-Casp3, Casp8, iNOS, and NOX1 expressions in NCM460 cells treated with propionate and TNF-α (Fig. 7D,E).

### Propionate alleviates NOX1-induced ROS injury of colonic epithelial cells dependent on HDAC2

**Fig. 8:**
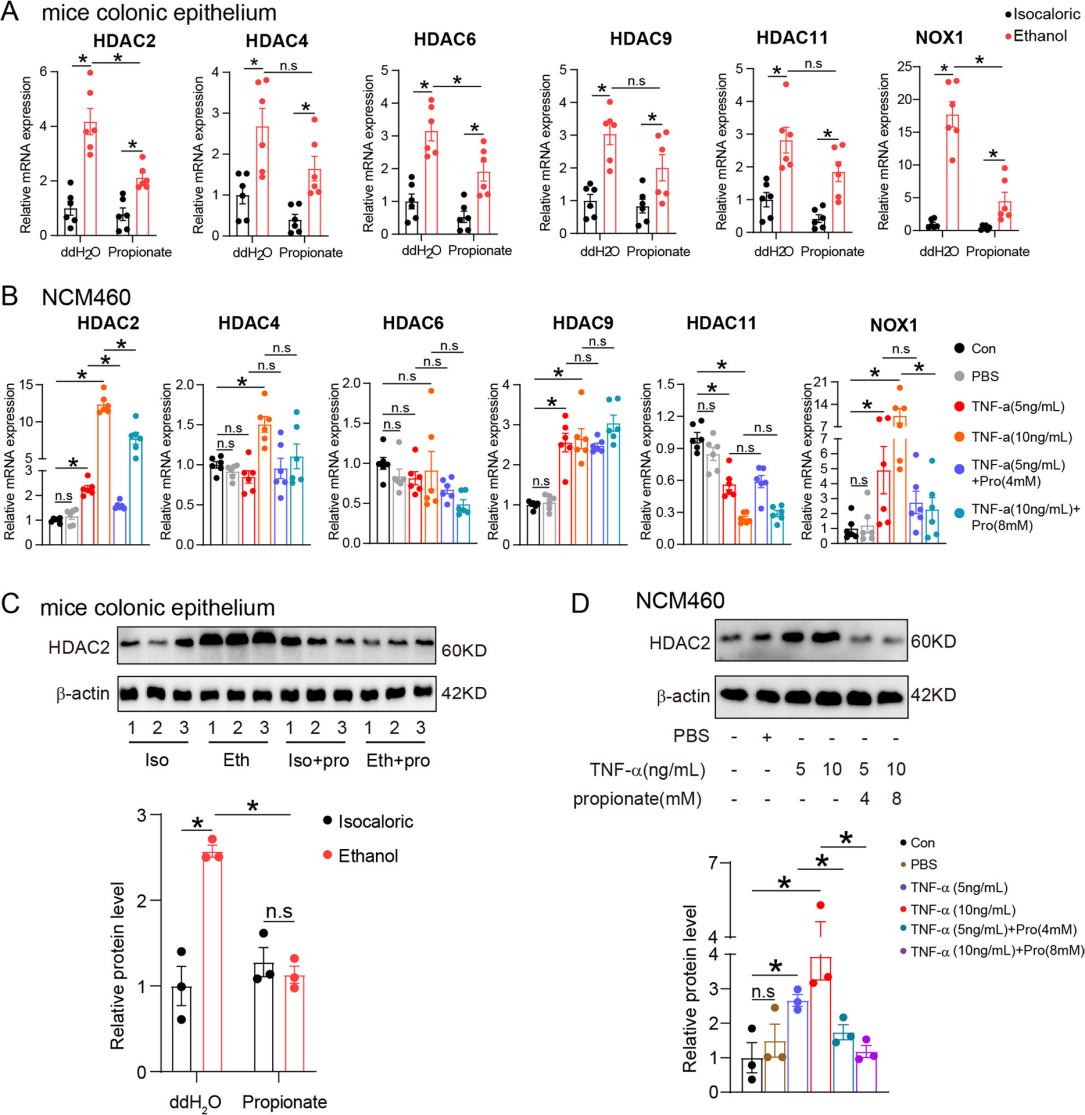
Propionate alleviating ROS injury of colonic epithelial cells was through inhibiting the expression of HDAC2. **A,** mRNA levels of HDAC2, HDAC4, HDAC6, HDAC9, HDAC11, and NOX1 in colonic epithelial cells isolated from isocaloric-fed and ethanol-fed mice with and without propionate supplement. *N* = 6 of each group. **B**, mRNA levels of HDAC2, HDAC4, HDAC6, HDAC9, HDAC11, and NOX1 in NCM460 cells treated with TNF-α and propionate. *N* = 6 for each group. **C**, HDAC2 protein expression of colonic epithelial cells isolated from isocaloric-fed and ethanol-fed mice with and without propionate supplement. *N* = 3 for each group. **D**, HDAC2 protein expression of NCM460 cells treated with TNF-α and propionate. *N* = 3 for each group. n.s, no significant. *p value < 0.05

Studies have shown the effects of SCFAs on intestinal inflammation via their HDAC-inhibiting ability (He et al. 2020; Visekruna and Luu 2021). Since the DEGs were enriched in histone deacetylase binding as shown through mRNA sequencing (Fig. 2D), we examined the expression of HDAC2, HDAC4, HDAC6, HDAC9, and HDAC11 in the colonic epithelial cells of mice and NCM460 cells. The levels of the above-mentioned HDACs were elevated in ethanol-exposed mice, compared with control mice, but only HDAC2 and HDAC6 were downregulated due to further propionate treatment (Fig. 8A). In the NCM460 cells treated with TNF-α, HDAC2, HDAC4, and HDAC9 expression levels increased, but that of HDAC6 did not increase compared with the control group (Fig. 8B). Surprisingly, HDAC11 expression was downregulated in NCM460 cells treated with TNF-α compared with the controls. Propionate supplement led to a significantly decreased HDAC2 expression in NCM460 cells but had no effects on HDAC4 and HDAC9 levels. NOX1 was highly expressed in alcohol-exposed colonic epithelial cells and TNF-α-treated NCM460 cells compared with control groups and downregulated by propionate treatment (Fig. 8A, B). The protein levels of HADC2 in colonic epithelial cells of the alcohol-exposed mice and NCM460 cells showed a similar trend, as shown through the immunoblotting assay (Fig. 8C, D). Therefore, we concluded that HDAC2 regulated the expression of NOX1.

**Fig. 9:**
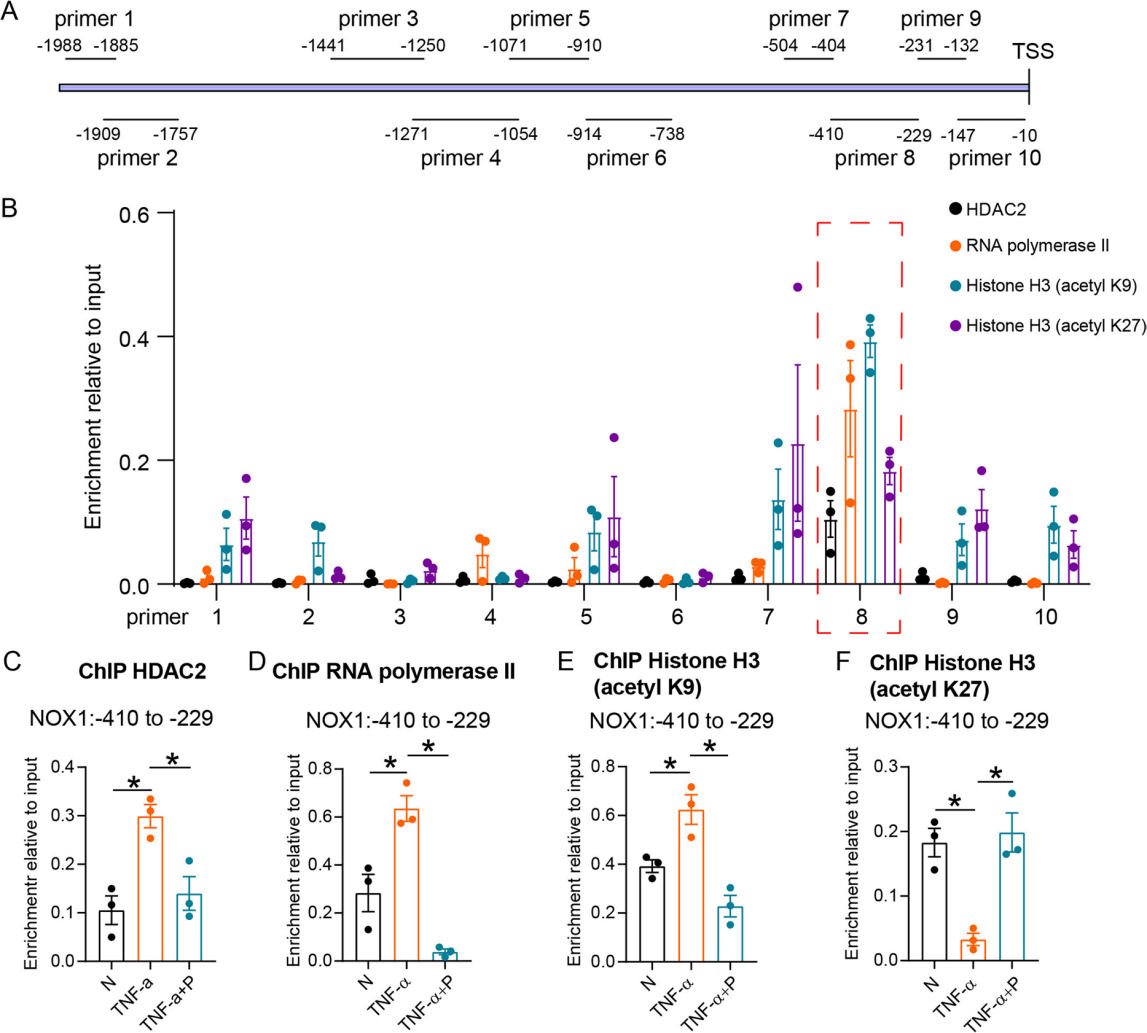
Propionate inhibited the expression of NOX1 by histone acetylation modification. **A**, Position of primers in the NOX gene promoter region (from 0 to 2000 bp upstream of transcriptional start site). **B**, Chromatin immunoprecipitated by HDAC2, RNA polymerase II, histone H3 (K9), and histone H3 (K27), and PCR analysis of the epigenetic modifying site of NOX1 promoter region using the 10 primers. **C**–**F**, Changes in HDAC2 (**C**), RNA polymerase II binding (**D**), histone H3 (K9) (**E**), and histone H3 (K27) (**F**) acetylation of NOX1 promoter region when NCM460 cells exposed to TNF-α and propionate (P). *N* = 3 for each group. *p value < 0.05

To investigate the mechanism of HADC2-mediated NOX1 gene transactivation, the cultured NCM460 cells were subjected to chromatin immunoprecipitation (ChIP) assays. We designed 10 pairs of primers, ranging from 0 to 2000 bp, upstream of the NOX gene transcriptional start site (Fig. 9A). ChIP-PCR indicated that HDAC2 and RNA polymerase II binding sites were located at the proximal promoter region of NOX1 (primer 8: − 410 to − 229 bp) (Fig. 9B). H3K27ac and H3K9ac were also enriched in upstream of the NOX1 gene promoter.

To assess the regulation effect of propionate on NOX1 expression through histone acetylation of the gene promoter region, cultured NCM460 cells were exposed to 10 ng/mL TNF-α with or without 8 mM propionate for 24 h and subjected to ChIP assay and RT-PCR analysis. The results showed that TNF-α induced an increase in HDAC2 and RNA polymerase II binding to the NOX1 promoter region, which decreased after propionate was added (Fig. 9C,D). Histone H3 (K9) acetylation in the NOX1 promoter region increased after TNF treatment and decreased after propionate supplement (Fig. 9E). Conversely, a significant level of reduction in levels of histone H3 (K27) acetylation in the NOX1 promoter region of TNF-α-treated NCM460 cells was observed, which increased after propionate was added (Fig. 9F).

## Discussion

Intestinal metabolites, the products of a mutually beneficial relationship between the host and its resident microbiota (Nicholson et al. 2012), are affected by chronic ethanol consumption. Dietary fiber intake is beneficial for constipation, insulin resistance, colonic inflammation, cardiovascular disease, and colorectal carcinoma prevention (Barber et al. 2020). Gut bacteria-derived SCFAs play a crucial role in modulating intestinal homeostasis and pathogen defense. SCFA-mediated signaling not only regulates energy metabolism (Canfora et al. 2019) but also plays an important role in gut immunity responses (Kasubuchi et al. 2015; Kim et al. 2013). Propionate has been reported to enhance the migration and polarization of intestinal epithelial cells to protect against ulcer formation in experimental colitis (Chu et al. 2020). Tong Ling-Chang et al. found that propionate could ameliorate dextran sodium sulfate-induced intestinal inflammation and oxidative stress (Tong et al. 2016). In a double-blinded, randomized placebo-controlled study, healthy volunteers with daily butyrate enemas that had persisted for two weeks were observed to show increased transcriptional regulation of the pathways indicating fatty acid oxidation and oxidative stress in sigmoid colon biopsy tissues compared with the placebo group (Hamer et al. 2009; Vanhoutvin et al. 2009). Our study found that propionate could prevent alcohol-related apoptosis of colonic epithelial cells. Gut leakiness and intestinal oxidative injury were preceded by alcoholic steatohepatitis (ASH) (Keshavarzian et al. 2009), indicating propionate may be an effective treatment agent for ASH.

Oxidative stress, which creates high levels of peroxynitrite and superoxide, results in the nitration and carbonylation of tight junctional and cytoskeletal proteins and apoptosis of enterocytes, which is responsible for alcohol-induced intestinal barrier injury (Cho et al. 2018). However, the mechanism by which alcohol induces oxidative stress in intestinal epithelial cells remains unclear. Researchers found that CYP2E1-mediated reactive oxygen species act as the main cause of ethanol-induced liver injury (Lu et al. 2010, 2008). Meanwhile, CYP2E1 potentiated binge alcohol-induced oxidative stress in the small intestine and the gut leakiness (Abdelmegeed et al. 2013). Other than CYP2E1, other oxidative enzymes, such as NADPH oxidase, are also critical in alcohol-caused liver injury. The p47phox (a critical regulatory subunit of NADPH oxidase) knockdown mice had decreased free radical production and hepatic inflammation and were alleviated from alcohol-induced liver injury (Kono et al. 2000). However, its role in gut permeability has not yet been explored. We found that NOX1 levels increased significantly in the colonic epithelial cells of chronic alcohol-exposed mice. Cremonini et al. have pointed out that a high-fat diet induces high levels of NOX1 and NOX4 expressions, accompanied by the activation of redox-sensitive signals (NF-κB and ERK1/2) and increased intestinal permeability (Cremonini et al. 2019). Therefore, NOX1-mediated oxidative stress is potentially an important cause of alcohol-related intestinal barrier function impairment. Our in vitro studies, which showed that NOX1 inhibitors alleviated oxidative stress injury in NCM460 cells, proved this point to a certain extent. In vivo intervention of NOX1 expression to verify its role in oxidative stress injury of enterocytes may be an interesting study.

SCFAs (especially propionate and butyrate) are known as endogenous HDACs inhibitors and epigenetically regulate the expression of targeted genes (He et al. 2020; Koh et al. 2016). In addition, SCFAs work through G protein-coupled receptors, mainly FFAR2 and FFAR3. SCFAs have been investigated as therapeutic agents that can attenuate inflammatory disorders and carcinogenesis based on their function of modulating immunometabolism and inhibiting HDAC (Visekruna and Luu 2021). We demonstrated that propionate-inhibited, alcohol-induced NOX1 expression in colonic epithelial cells was independent of G protein-coupled receptors (FFAR2 and FFAR3), but dependent on HDAC2-induced change of the histone acetylation status of the NOX1 promoter region.

Researchers have shown that NOX1 expression is regulated by gene histone acetylation modification. Pharmacological inhibition of HDAC downregulates NOX1, NOX4, and NOX5 expression levels and NADPH-stimulated ROS formation in the aortas of diabetic mice (Manea et al. 2018), while HDAC inhibitors reduced NOX4 expression and H_2_O_2_ formation in the vascular endothelial cells (Hakami et al. 2016). Interestingly, H4K16 acetylation mediated NOX gene transactivation in the macrophages of ischemic and reperfusion heart patients (Yu et al. 2018). We performed ChIP-PCR assays to gain more insights into the interactions between the NOX1 gene promotor region and HDAC2. Physical interactions between HDAC2 in the active transcription region of NOX1 and RNA polymerase II were observed. H3K9ac enrichment was high in TNF-α exposed NCM460 cells but decreased after propionate supplementation. However, H3K27ac showed an opposite trend. In general, histone acetylation promotes gene transcription, but different sites of histone acetylation form different cooperative networks in a context-dependent manner to regulate gene expression differently. Mass spectrometry can be conducted on histone acetylation in future research studies to help explain the cooperative network. From the data presented thus far, it can be observed that propionate acts as an HDAC inhibitor to intelligently regulate the histone acetylation of NOX1 and powerfully alleviate ethanol-related ROS injury in the colon. Antioxidants have been found protective in ALD (Han et al. 2020; Neha et al. 2019). Other natural antioxidants such as sulforaphane, epigallocatechin gallate, and plumbagin might be a powerful therapy for ALD (Marrazzo et al. 2019).

## Conclusion

In conclusion, we found that alcohol induces the significant upregulation of NOX1 and ROS accumulation in colonic epithelial cells that results in the apoptosis of enterocytes and damage to the intestinal barrier. Propionic acid, but not the other SCFAs, was reduced in the cecum of chronic–binge ethanol-fed mice in comparison to controls. Propionate supplementation reduced levels of ROS production and the apoptosis of colonic epithelial cells and alleviated liver injury. Propionate exerted its effect by reducing NOX1 expression in colonic epithelial cells through HDAC2 inhibition but not through the free fatty acid receptors, FFAR2 and FFAR3.

### Supplementary Information

Below is the link to the electronic supplementary material.

Supplementary file1 (DOCX 785 KB)

## Data Availability

Data that support the results of this study have been shown in the manuscript and Supplementary information. More data are available on request from the authors.
