# Supplementary material for: The role of NADPH oxidase 1 in alcohol-induced oxidative stress injury of intestinal epithelial cells
Source: Cell Biol Toxicol. 2022 May 31;39(5):2345–64. doi: 10.1007/s10565-022-09725-1 (PMC10547661; doi:10.1007/s10565-022-09725-1)
Supplement: Supplementary file 1 — Supplementary file1 (DOCX 785 KB) [file 10565_2022_9725_MOESM1_ESM.docx]

Role of NADPH oxidase 1 in alcohol-caused oxidative stress injury of intestinal epithelial cells

Liuying Chen^1^, Huikuan Chu^1^, Lilin Hu^1^, Zhonglin Li^1^, Ling Yang^1#^, Xiaohua Hou^1#^

Liuying Chen, Email: jinniuzuo400@126.com

Huikuan Chu, Email: 2012xh0827@hust.edu.cn

Lilin Hu, Email: hulilin95@163.com

Zhonglin Li, Email: 1479590989@qq.com

Ling Yang, Email: hepayang@163.com

Xiaohua Hou, Email: houxh@hust.edu.cn

^1^Division of Gastroenterology, Union Hospital, Tongji Medical College, Huazhong University of Science and Technology, 1277 Jiefang Avenue, Wuhan 430022, China

**^#^Correspondence**

Xiao Hua Hou, M.D., Ph.D. Email: houxh@hust.edu.cn; Division of Gastroenterology, Union Hospital, Tongji Medical College, Huazhong University of Science and Technology, 1277 Jiefang Avenue, Wuhan, 430022, China; Phone +86 -2785726678, +8613035143646.

Ling Yang, M.D., Ph.D. Email: hepayang@163.com; Division of Gastroenterology, Union Hospital, Tongji Medical College, Huazhong University of Science and Technology, 1277 Jiefang Avenue, Wuhan, 430022, China; phone +86-2785726678, +8613971178791.

Supplemental table 1. Primers used in RT-PCR.

| Gene name | Forward sequence | Reverse sequence |
| --- | --- | --- |
| Mouse 18S | GTAACCCGTTGAACCCCATT | CCATCCAATCGGTAGTAGCG |
| Total bacteria | GTGSTGCAYGGYTGTCGTCA | ACGTCRTCCMCACCTTCCTC |
| Mouse IL-1β | TTGTTGATGTGCTGCTGTGA | TGTGAAATGCCACCTTTTGA |
| Mouse TGF-β | GGCACCATCCATGACATGAACC | CCGCACACAGCAGTTCTTCTCT |
| Mouse IL-6 | CTGCAAGAGACTTCCATCCAG | AGTGGTATAGACAGGTCTGTTGG |
| Mouse TNF-α | CTGAACTTCGGGGTGATCGG | GGCTTGTCACTCGAATTTTGAGA |
| Mouse MCP-1 | TTAAAAACCTGGATCGGAACCAA | GCATTAGCTTCAGATTTACGGGT |
| Mouse IFN-γ | ATGAACGCTACACACTGCATC | CCATCCTTTTGCCAGTTCCTC |
| Mouse HDAC2 | ATGGCGTACAGTCAAGGAGG | ATGAGGCTTCATGGGATGACC |
| Mouse HDAC4 | CACTGCATTTCCAGCGATCC | AAGACGGGGTGGTTGTAGGA |
| Mouse HDAC6 | GAGGAGCTGATGTTGGTTCAC | AGTTCGGATGCAGATACACTGA |
| Mouse HDAC9 | GCGGTCCAGGTTAAAACAGAA | GCCACCTCAAACACTCGCTT |
| Mouse HDAC11 | GTGTACTCACCACGTTACAACA | GCTCGTTGAGATAGCGCCTC |
| Mouse NOX1 | GGTTGGGGCTGAACATTTTTC | TCGACACACAGGAATCAGGAT |
| Mouse Gpx1 | AGTCCACCGTGTATGCCTTCT | GAGACGCGACATTCTCAATGA |
| Mouse Sod1 | AACCAGTTGTGTTGTCAGGAC | CCACCATGTTTCTTAGAGTGAGG |
| Mouse Cat | AGCGACCAGATGAAGCAGTG | TCCGCTCTCTGTCAAAGTGTG |
| Human GAPDH | GGAGCGAGATCCCTCCAAAAT | GGCTGTTGTCATACTTCTCATGG |
| Human  HDAC2 | ATGGCGTACAGTCAAGGAGG | TGCGGATTCTATGAGGCTTCA |
| Human  HDAC4 | CACGCACAGTCCTTGGTTGGT | TGCTGATGCTGCTGCTGGATG |
| Human  HDAC6 | ACCATCCAAGTCCATCGCAGAT | TCGTGTGGTCATCCGCTCAG |
| Human  HDAC9 | AGTAGAGAGGCATCGCAGAGA | GGAGTGTCTTTCGTTGCTGAT |
| Human  HDAC11 | ACCCAGACAGGAGGAACCATA | TGATGTCCGCATAGGCACAG |
| Human  NOX1 | CTGCTTCCTGTGTGTCGCAA | AGGCAGATCATATAGGCCACC |
| Human  FFAR2 | CCGTGCAGTACAAGCTCTCC | CTGCTCAGTCGTGTTCAAGTATT |
| Human  FFAR3 | TTCACCACCATCTATCTCACCG | GGAACTCCAGGTAGCAGGTC |
| Human  Gpx1 | CAGTCGGTGTATGCCTTCTCG | GAGGGACGCCACATTCTCG |
| Human  Sod1 | GGTGGGCCAAAGGATGAAGAG | CCACAAGCCAAACGACTTCC |
| Human  Cat | TGGGATCTCGTTGGAAATAACAC | TCAGGACGTAGGCTCCAGAAG |

Supplemental table 2. Primers used in CHIP-PCR.

| Gene name | Forward sequence | Reverse sequence |
| --- | --- | --- |
| NOX1 primer1 | ACTCGGGTGGCTAAGGCATGA | GGTCTCACTCTGTCACCCAGGT |
| NOX1 primer2 | CCAACCTGGGTGACAGAGTGA | TTGAATCCACCTGGAGCTGATT |
| NOX1 primer3 | TAGGCTCAGAATAGGCTCTT | CTTCCTCTACATGCTTACCAA |
| NOX1 primer4 | GTTGGTAAGCATGTAGAGGA | CTACTGATGGATATGATGATGG |
| NOX1 primer5 | CATCATATCCATCAGTAGTAGG | TGTGTATGTATGCTTCTGTG |
| NOX1 primer6 | TGAAGGGAGATCACACCACTG | TGAATCCACCTGGAGCTGATT |
| NOX1 primer7 | GAGTGCTCTGCCTCTGTT | GGCTGGAAGGACAAGTGA |
| NOX1 primer8 | TCCAGCCAGTCAAGTTCTCC | GGAAGCGTGTGTGGTAAGGA |
| NOX1 primer9 | TCCCTCCCTACTTCTCCT | AGCATTGCCTTCCTAGATAA |
| NOX1 primer10 | CTAGGAAGGCAATGCTTCACAT | CCAGCCCTATCTATGAGAACCA |


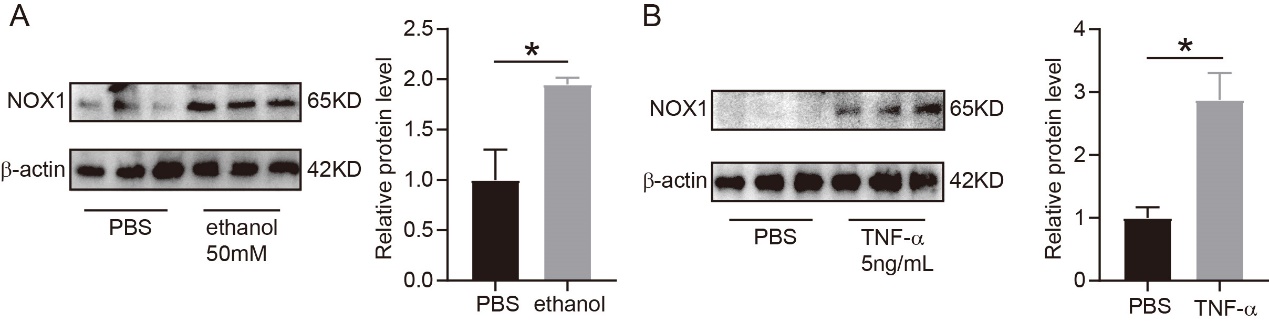


Supplemental figure 1. NOX1 expression of NCM460 cells treated with ethanol and TNF-α. A. NOX1 expression of NCM460 cells treated with ethanol (50mM) for 24h. B. NOX1 expression of NCM460 cells treated with TNF-α(5ng/mL) for 24h. N=3 of each group. *indicated p<0.05.

Supplemental figure 2. Inflammatory factors expression of colonic tissues from mice isocaloric-fed and ethanol-fed mice with and without propionate supplement by RT-PCR. N=6 of each group. n.s, no significant. *indicated p<0.05.


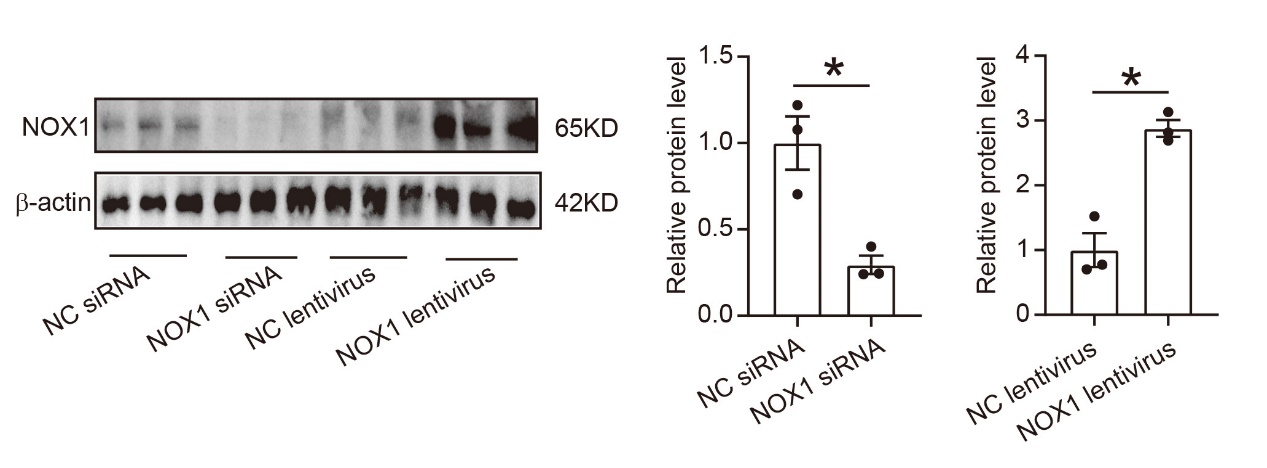


Supplemental figure 3. Evaluation of NOX1 intervention and overexpression by western blotting. N=3 of each group. *indicated p<0.05.

Supplemental figure 4. Propionate alleviated ROS injury of NCM460 cells treated with TNF-α. A. Tunel staining of NCM460 cells exposed to TNF-α with and without propionate. B, C. DCFH-DA fluorescent probe assay (B) and DHE staining (C) showed ROS formation in NCM460 cells from above groups. E. Immunoblotting images and quantification of Bax, Casp3, c-Casp3, Casp8, iNOS and NOX1 protein expressions from the above groups. N=3 of each group. n.s, no significant. *p value<0.05.


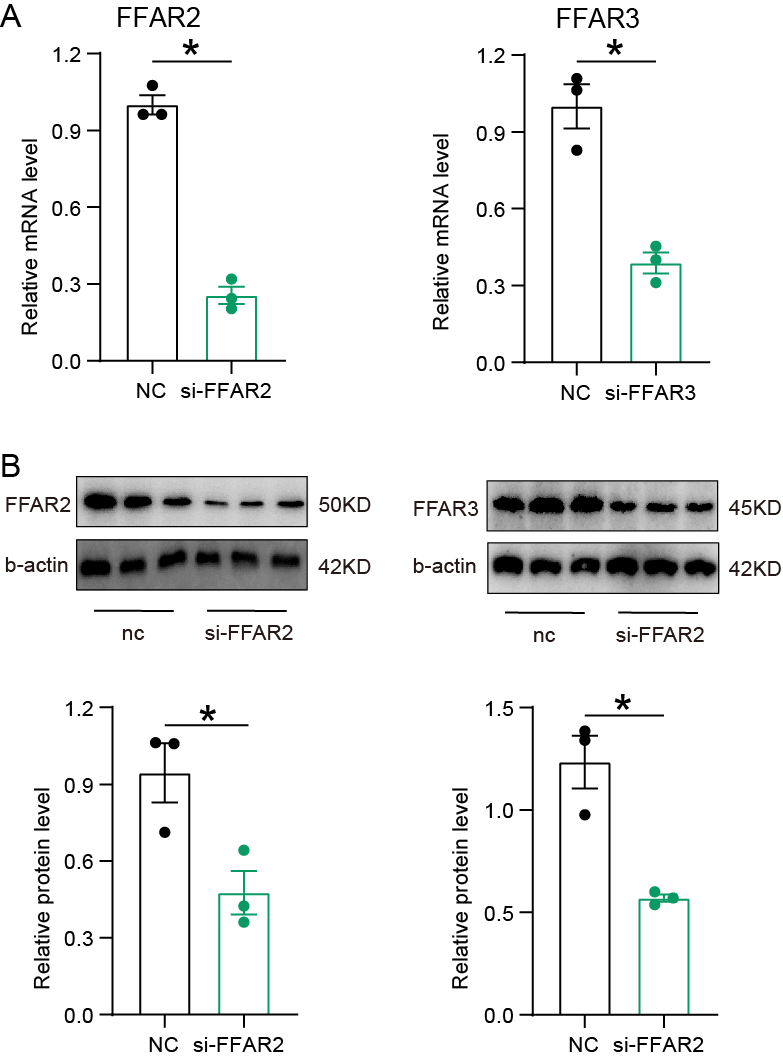


Supplemental figure 5. Effect of siRNA intervention. A. mRNA levels of FFAR2 and FFAR3 after siRNA interfering NCM460 cells. B. Protein levels of FFAR2 and FFAR3 after siRNA interfering NCM460 cells. N=3 of each group. *p value<0.05.
